# Association of prediabetes-associated single nucleotide polymorphisms with microalbuminuria

**DOI:** 10.1371/journal.pone.0171367

**Published:** 2017-02-03

**Authors:** Jong Wook Choi, Shinje Moon, Eun Jung Jang, Chang Hwa Lee, Joon-Sung Park

**Affiliations:** 1 Department of Internal Medicine, Hanyang University College of Medicine, Seoul, South Korea; 2 Division of Endocrinology and Metabolism, Hallym University College of Medicine, Seoul, South Korea; 3 Korea Centers for Disease Control and Prevention, Cheongju-si, Chungcheongbuk-do, Korea; Medical Clinic, University Hospital Tuebingen, GERMANY

## Abstract

Increased glycemic exposure, even below the diagnostic criteria for diabetes mellitus, is crucial in the pathogenesis of diabetic microvascular complications represented by microalbuminuria. Nonetheless, there is limited evidence regarding which single nucleotide polymorphisms (SNPs) are associated with prediabetes and whether genetic predisposition to prediabetes is related to microalbuminuria, especially in the general population. Our objective was to answer these questions. We conducted a genomewide association study (GWAS) separately on two population-based cohorts, Ansung and Ansan, in the Korean Genome and Epidemiology Study (KoGES). The initial GWAS was carried out on the Ansung cohort, followed by a replication study on the Ansan cohort. A total of 5682 native Korean participants without a significant medical illness were classified into either control group (n = 3153) or prediabetic group (n = 2529). In the GWAS, we identified two susceptibility loci associated with prediabetes, one at 17p15.3-p15.1 in the *GCK* gene and another at 7p15.1 in *YKT6*. When variations in *GCK* and *YKT6* were used as a model of prediabetes, this genetically determined prediabetes increased microalbuminuria. Multiple logistic regression analyses revealed that fasting glucose concentration in plasma and SNP rs2908289 in *GCK* were associated with microalbuminuria, and adjustment for age, gender, smoking history, systolic blood pressure, waist circumference, and serum triglyceride levels did not attenuate this association. Our results suggest that prediabetes and the associated SNPs may predispose to microalbuminuria before the diagnosis of diabetes mellitus. Further studies are needed to explore the details of the physiological and molecular mechanisms underlying this genetic association.

## Introduction

Diabetes mellitus (DM) and its vascular complications have become global socioeconomic and public health problems [1, 2]. Diabetic kidney disease (DKD), one of the most common microvascular complications of DM, seems to increase the risk of cardiovascular mortality [3, 4]. Thus, early identification of potential risk factor(s) of DKD and a preventive strategy against DKD are crucial for improvement of long-term health and survival.

Prediabetes, which refers to a plasma glucose level that is above the normal range but not high enough to meet the diagnostic criteria of DM, usually indicates a risk of conversion to type 2 DM (T2D) [5–7]. Even though not all patients with prediabetes progress to full-blown T2D, recent epidemiological studies have shown that subjects with prediabetes have various forms of vascular complications associated with T2D before the diagnosis of DM, which are also associated with an increased risk of kidney disease and cardiovascular morbidity and mortality [6–10]. Such findings suggest that even prediabetes may be a leading cause of complications that are typically attributed to DM.

Microalbuminuria, small amounts of albumin leakage into urine, indicates dysfunction of the glomerular filtration barrier, which is not only the early feature of a diabetic microvascular complication but also an independent risk factor of cardiovascular disease, even in nondiabetic populations [11–13]. In addition to the reports about the association between prediabetes and microalbuminuria, there have been many studies that reveal genetic variations associated with susceptibility to proteinuria in patients with T2D [14–19]. Such findings suggest that a complex interaction of genetic and environmental factors may have positive or negative influence(s) on both hyperglycemia and the related complications. Nonetheless, there is only limited evidence showing how genetic and nongenetic determinants of prediabetes may interact with microalbuminuria. Our aim was to clarify the association of prediabetes with microalbuminuria in the general population. Therefore, we conducted a genomewide association study (GWAS), which yielded useful results.

## Results

### The relation between prediabetes and microalbuminuria

The characteristics of each cohort and the study design are shown in Table 1 and S1 Fig. Out of the 5682 people included in the study, 2529 subjects had a diagnosis of prediabetes on the basis of fasting plasma glucose, 2-hour glucose in the oral glucose tolerance test, and glycated hemoglobin (HbA1c). The anthropometric, clinical, and laboratory details of the study participants—who were classified into two groups according to whether they had prediabetes—are shown in Table 2. Only urinary albumin-to-creatinine ratios (UACRs) were log-transformed because all the biomarkers except UACR appeared to be normally distributed. Mean fasting glucose, mean postprandial glucose, and HbA1c in the prediabetic group were all higher than those in subjects with a normal glucose level. In comparison with the controls, an increase UACR was prominent in subjects with prediabetes (2.4 ± 0.6 vs. 2.5 ± 0.7, *P* < 0.0001 after log transformation). Participants in the prediabetic group were older and more obese, had higher blood pressure (BP), and a worse lipid profile than the control group did. In particular, a mildly increased C-reactive protein (CRP) concentration in plasma and a decreased kidney function were observed in the prediabetic group.

**Table 1:** Characteristics of the study population.

Stage	Study	Sample type	Source	Number of samples	Males (%)	Age (years)
GWAS	Ansung	Control	Korean Biobank Network	1362	589 (43)	52.6 ± 8.8
		Prediabetic	Korean Biobank Network	1092	494 (45)	55.5 ± 8.6
Replication	Ansan	Control	Korean Biobank Network	1791	874 (49)	46.4 ± 6.2
		Prediabetic	Korean Biobank Network	1437	761 (53)	48.7 ± 7.6
Combined	Ansung + Ansan	Control	Korean Biobank Network	3153	1463 (46)	49.1 ± 8.0
		Prediabetic	Korean Biobank Network	2529	1690 (50)	51.7 ± 8.7

**Table 2:** Baseline characteristics grouped according to case-control status.

	Prediabetic state		p
	Control (n = 3153)	Case (n = 2529)	
Age (year)	49.1 ± 8.0	51.7 ± 8.7	<0.0001
Gender (male, %)	1463 (46)	1255 (50)	0.0156
Systolic BP (mmHg)	116.4 ± 12.1	118.5 ± 12.1	<0.0001
Diastolic BP (mmHg)	79.0 ± 9.7	80.4 ± 9.6	<0.0001
Body mass index (kg/m^2^)	23.9 ± 2.7	24.6 ± 3.1	<0.0001
Waist circumference (cm)	80.0 ± 8.3	82.3 ± 8.6	<0.0001
eGFR^a^, mL/(min·[1.73 m^2^])	77.7 ± 12.6	76.7 ± 12.8	0.0044
eGFR < 60 (n, %)	93 (3)	157 (6)	<0.0001
Hemoglobin (g/L)	135 ± 16	136 ± 16	0.1676
Albumin (g/L)	42.3 ± 3.1	42.6 ± 3.4	0.0042
Fasting glucose (mmol/L)	4.45 ± 0.37	4.72 ± 0.52	<0.0001
Postprandial glucose (mmol/L)	5.63 ± 1.13	7.09 ± 1.81	<0.0001
Hemoglobin A1c (%)	5.33 ± 0.22	5.89 ± 0.19	<0.0001
Triglycerides (mmol/L)	1.57 ± 0.98	1.84 ± 1.21	<0.0001
HDL-cholesterol (mmol/L)	1.18 ± 0.26	1.16 ± 0.35	0.0300
LDL-cholesterol (mmol/L)	2.85 ± 0.8	3.03 ± 0.85	<0.0001
C-reactive protein (nmol/L)	1.71 ± 3.52	2.29 ± 4.86	<0.0001
UACR (mg/[g Cr])	14.5 ± 16.6	16.6 ± 20.5	0.0104
Log-UACR (log mg/[g Cr])	2.4 ± 0.6	2.5 ± 0.7	<0.0001
Smoking history (%)			<0.0001
• Never smoker	1932 (61)	1406 (56)	
• Ex-smoker	433 (14)	408 (16)	
• Intermittent smoker	100 (3)	67 (3)	
• Chain smoker	688 (22)	648 (26)	

Results are expressed as mean ± SD or as frequencies (and proportions).

BP, blood pressure; eGFR, estimated glomerular filtration rate; LDL, low-density lipoprotein; Log-UACR, log-transformed urine albumin/creatinine ratio; Cr, creatinine.

^a^estimated using the Chronic Kidney Disease Epidemiology Collaboration (CKD-EPI) equation.

**Fig 1:**
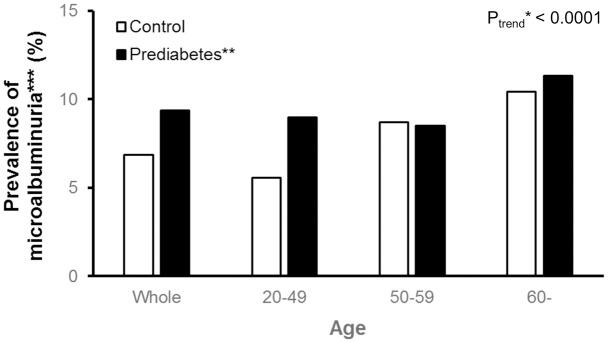
Age-specific prevalence of microalbuminuria by prediabetic state. *calculated by the Cochran-Armitage test for a trend. **defined as fasting glucose between 5.6 and 7.0 mmol/L, postprandial glucose between 7.8 and 11.0 mmol/L, or glycated hemoglobin between 5.7% and 6.4%. ***defined as a urine albumin/creatinine ratio (mg/[g creatinine]) between 30 and 300.

In the multiple linear regression analysis with adjustment for age, gender, body mass index and waist circumference, log-UACR had a strong positive relation with fasting blood glucose levels (β = 0.0040, *r*^2^ = 0.1257, *P* = 0.0479) and HbA1c (β = 0.1274, *r*^2^ = 0.1276, *P* = 0.0059). Furthermore, with the trend of increasing prevalence of microalbuminuria with age (P for the trend <0.0001), we found that the prediabetic state was related to increased prevalence of microalbuminuria especially in younger subjects (Fig 1 and data not shown).

### GWAS on prediabetes

We analyzed genetic data from 1092 participants in a prediabetic state (case) and 1362 healthy people (control) from the Ansung cohort. We carried out logistic regression analysis for prediabetes by including age and gender as covariates, and calculated the minimal p value for three genetic models (additive, recessive, and dominant). After a standard quality control procedure, we obtained genotyping results on 1,198,063 SNPs and generated a quantile-quantile plot (S2 Fig). The genomic inflation factor λ was 1.01654 in the quantile-quantile plot, indicating minimal evidence of population stratification. The association analysis revealed that a total of 20 SNPs from 17 distinct genomic regions were significantly associated with prediabetes in the Ansung cohort (p_GWAS_ ranging from 9.54 × 10^−5^ to 4.29 × 10^−6^, S1 Table).

#### Replication stage and combined analysis

We performed subsequent association analysis of genetic data from the Ansan cohort on the basis of the 20 aforementioned SNPs and found significant associations for SNPs rs2908289, rs1799884, and rs917793 (p_Replication_ ranging from 9.79 × 10^−5^ to 8.97 × 10^−6^, S2 Table). Finally, these top three SNPs were used for combined analysis, which showed that prediabetes was significantly associated with a genetic polymorphism at 7p15 in the three genetic models (rs2908289, p_Meta_ = 2.5 × 10^−7^; rs1799884, p_Meta_ = 1.7 × 10^−7^; rs917793, p_Meta_ = 4.6 × 10^−8^; Table 3).

**Table 3:** Analysis of the association of the top three single nucleotide polymorphisms (SNPs) with prediabetes.

dbSNP ID	Nearest gene	Genotype	Study	Genotype frequency (%)			Additive^a^		Dominant^a^		Recessive^a^	
		1/2/3	state	1	2	3	OR	p	OR	p	OR	p
rs2908289	*GCK/LOC105375257*	AA/AG/GG	Control	69.3	28.0	2.7	1.40	4.5 × 10^−6^	1.34	2.5 × 10^−7^	1.82	4.3 × 10^−5^
			Case	62.8	32.4	4.8						
rs1799884	*GCK*	TT/TC/CC	Control	69.1	28.1	2.8	1.41	4.2 × 10^−6^	1.35	1.7 × 10^−7^	1.82	4.3 × 10^−5^
			Case	62.6	32.6	4.8						
rs917793	*YKT6*	TT/TA/AA	Control	65.6	30.4	4.0	1.35	2.3 × 10^−6^	1.36	4.6 × 10^−8^	1.67	4.8 × 10^−5^
			Case	58.5	35.1	6.3						

OR, Odds ratio.

^a^calculated by logistic regression analysis with age and gender as covariates.

**Fig 2:**
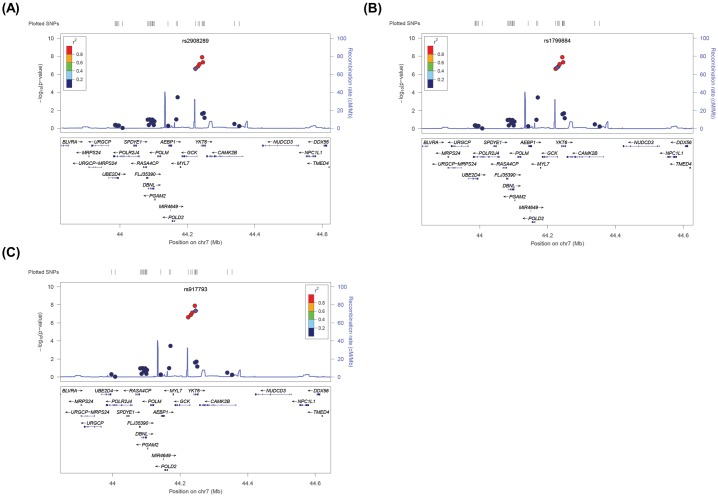
A regional association plot of SNPs from chromosome 7 associated with prediabetes. Data on the associated region on chromosome 7 include (A) rs2908289 (in *GCK*), (B) rs1799884 (in *GCK*), and (C) rs917793 (in *YKT6*). The p values of genotyped SNPs are plotted as −log_10_ values against their physical position on each chromosome (NCBI Build 36/hg19).

Because the SNPs of interest were located in a genomic region encoding, for example, *POLM*, *AEBP1*, *MYL7*, *GCK*, *YKT6*, and *CAMK2B*, we conducted an imputation analysis to characterize these loci (Fig 2). The regional association plots using genotyped and imputed data revealed that rs2908289 and rs1799884 are confined to regions around the *GCK* gene encoding glucokinase, and rs917793 is located in the *YKT6* gene encoding YKT6 (v-SNARE homolog).

### Prediabetes-associated SNPs and microalbuminuria

In Mendelian randomization, which is a form of instrumental variable regression analysis, the instrumental variable-estimated size of the effect of prediabetes on log-UACR was highly significant and consistent for all outcomes (β = 0.015 [95% confidence interval (CI) 0.014–0.016] for fasting plasma glucose, 0.065 [0.011–0.012] for postprandial glucose, and 0.216 [0.198–0.234] for HbA1c; Table 4). We performed subsequence analysis for possible associations between candidate genetic polymorphisms and the clinical and laboratory characteristics of all the participants. Multiple logistic analysis adjusted for age and gender indicated that fasting glucose levels and SNPs in the *GCK* and *YKT6* genes were associated with microalbuminuria, and further adjustment for conventional risk factors, such as age, gender, smoking history, systolic BP, waist circumference, and serum triglyceride level, showed that the genetic polymorphism at rs2908289 was significantly associated with microalbuminuria, especially in recessive models (Table 5).

**Table 4:** Comparison of the association of prediabetes with Log-UACR obtained from ordinary least squares linear regression to that obtained from the instrumental variables regression analysis^a^.

Outcome	β for SNP in age- and gender-standardized Log-UACR				Endogeneity p^b^
	Ordinary least squares linear regression		Instrumental variables analysis^a^		
	β (95% *CI*)	p	β (95% *CI*)	p	
Fasting glucose (mmol/L)	0.005 (0.003–0.007)	0.0195	0.015 (0.014–0.016)	<0.0001	<0.0001
Postprandial glucose (mmol/L)	0.000 (0.000–0.000)	0.5683	0.011 (0.010–0.012)	<0.0001	<0.0001
Hemoglobin A1c (%)	0.101 (0.057–0.145)	0.0223	0.216 (0.198–0.234)	<0.0001	<0.0001

^a^In instrumental variables regression analysis, *GCK* polymorphism rs2908289 and rs1799884, *YKT6* polymorphism rs917793 act as instruments for the effect of prediabetes on Log-UACR.

^b^Endogeneity was assessed by Durbin-Wu-Hausman test and reflects whether the difference in effect size between the two analytic approaches was statistically significant.

**Table 5:** Multivariate logistic regression analysis of microalbuminuria^a^.

Variable	Model I		Model II		Model III	
	OR	95% *CI*	OR	95% *CI*	OR	95% *CI*
Systolic BP (mmHg)	1.012	1.012–1.044	1.025	1.008–1.041		
Diastolic BP (mmHg)	1.023	1.003–1.043	1.019	0.999–1.039		
Body mass index (kg/m^2^)	1.072	1.013–1.135	1.054	0.992–1.120		
Waist circumference (cm)	1.031	1.011–1.051	1.026	1.005–1.047		
Hemoglobin (g/L)	0.985	0.851–1.141				
Albumin (g/L)	1.238	0.526–2.914				
eGFR, mL/(min·[1.73 m^2^])	0.995	0.873–1.135				
Fasting glucose (mmol/L)	1.028	1.008–1.049	1.026	1.005–1.048	1.030	1.007–1.054
Postprandial glucose (mmol/L)	1.003	0.998–1.009				
Hemoglobin A1c (%)	1.472	0.894–2.424				
Triglyceride (mmol/L)	1.002	1.001–1.003				
HDL-cholesterol (mmol/L)	1.007	0.992–1.023				
LDL-cholesterol (mmol/L)	1.010	0.998–1.015				
C-reactive protein (nmol/L)	1.28	0.989–1.655				
Smoking (vs. non-smoker)						
Ex-smoker	1.334	0.653–2.727				
Intermittent smoker	2.888	1.314–6.349				
Chain smoker	1.292	0.697–2.396				
rs2908289						
Additive model	1.340	1.021–1.759	1.409	1.067–1.862	1.262	0.922–1.729
Dominant model	1.214	0.874–1.686				
Recessive model	2.875	1.531–5.399	3.227	1.693–6.152	2.568	1.210–5.453
rs1799884						
Additive model	1.336	1.018–1.754	1.404	1.063–1.855	1.258	0.919–1.723
Dominant model	1.209	0.870–1.680				
Recessive model	2.871	1.529–5.393	3.223	1.691–6.144	2.567	1.209–5.451
rs917793						
Additive model	1.222	0.942–1.584				
Dominant model	1.148	0.831–1.587				
Recessive model	1.937	1.072–3.498	2.107	1.156–3.840	1.849	0.920–3.714

Model I: adjusted for age and gender.

Model II: adjusted for age, gender, smoking history, and serum triglyceride levels.

Model III: adjusted for age, gender, smoking history, systolic BP, waist circumference, and serum triglyceride levels.

OR, Odds ratio; *CI*, confidence interval.

^a^defined as a UACR between 30 and 300.

To determine the effect of different genotypes of rs2908289 on the possible risk factors of microvascular complications, each genotype was assessed by analysis of covariance and distinguished by the least significant difference method (Table 6). In addition to laboratory indicators of prediabetes, SNP rs2908289 showed a significant dose-dependent relation with microalbuminuria and serum triglyceride levels.

**Table 6:** Association of different genotypes of rs2908289 with microalbuminuria and its risk factors.

Variable	AA (n = 3762)		AG (n = 1705)		GG (n = 204)		p^a^
	Mean^a^	95% *CI*^a^	Mean^a^	95% *CI*^a^	Mean^a^	95% *CI*^a^	
eGFR, mL/(min·[1.73 m^2^])	77.1	76.7–77.4	77.7	77.2–78.2	77.0	75.5–78.5	0.1197
Fasting glucose (mmol/L)	4.54	4.52–4.56	4.58	4.56–4.61	4.62	4.55–4.70	0.0052
Postprandial glucose (mmol/L)	6.23	6.17–6.29	6.34	6.25–6.43	6.54	6.27–6.82	0.0179
Hemoglobin A1c (%)	5.50	5.49–5.51	5.53	5.51–5.55	5.62	5.56–5.67	<0.0001
Log-UACR (log mg/[g Cr])	2.42	2.37–2.46	2.45	2.34–2.51	2.61	2.44–2.79	0.0272

^a^estimated using analysis of covariance after adjustment for age, gender, smoking history, systolic BP, waist circumference, and serum triglyceride levels, and their differences were estimated by the least significant difference method.

## Discussion

In this study examining the association between prediabetes genetic predisposition and microalbuminuria, we demonstrated that SNP rs2908289 located in the GCK gene can predict the risk of renal microvascular complications associated with DM in the general population. Our results suggest that genetic components of the pathogenesis of prediabetes may be linked with susceptibility to diabetic vascular complications.

Chronic glycemic exposure is one of the important causes of micro- or macroangiopathy in patients with DM [20]. An increase in oxidative stress, followed by activation of various signaling pathways, has been reported to cause endothelial dysfunction and inhibition of vascular protective mechanisms in the hyperglycemic milieu [21, 22]. Some authors have argued that hyperglycemia-induced microvascular changes may be evident before the onset of DM, can be aggravated during the natural course of the disease, and eventually may be related to the increased risk of poor renal or cardiovascular outcomes [8–10]. Nonetheless, there is limited evidence supporting a direct relation between prediabetes and albuminuria as markers of diabetic microvascular complications [14–16]. In this study, we showed that there is a close association between the plasma glucose level and urinary excretion of albumin in the general population, suggesting that even prediabetes may play a possible role in the pathogenesis of microvascular complications. It was also found that the prediabetic group was associated with increased prevalence of microalbuminuria in young subjects in contrast to the control group, with the overall tendency of increasing microalbuminuria with age in both groups, in line with studies on microalbuminuria in the general population [23, 24]. Additionally, there were fewer people between the ages of 50 and 59 years in the prediabetes group than in the control group. This result is probably due to the exclusion of individuals with T2D in the study design.

In the initial GWAS analysis, we found that SNPs in the *GCK* gene are independently associated with prediabetes in the general population. GCK, the cytoplasmic subtype of hexokinase that catalyzes the initial step of several glucose metabolic pathways, acts as a gatekeeper for glucose-induced activation or deactivation of biological processes [22, 25]. Thus, regulation of its enzymatic activity is important for glucose homeostasis. In agreement with these data, some genetic studies have shown that mutations in the gene encoding GCK may be significantly associated with both hyperinsulinemic hypoglycemia and maturity onset diabetes of the young [26]. Moreover, subjects with mutations in the gene encoding GCK have lifelong mild hyperglycemia from birth, and this variant is significantly associated with progression to prediabetes or diabetes among the subjects with normal glucose tolerance [27–30]. Given that most of the existing studies were not confined to generally healthy subjects, it has been difficult to conclude that this genetic variation is directly associated with isolated prediabetes. Our results indicate that genetic variation of candidate genes may be associated with the initiation of aberrant glucose homeostasis in the general population.

The polymorphisms in the genetic loci within 7p15.3-p15.1 (encoding GCK) were found to be independently associated with an increased risk of microalbuminuria in subsequent analyses. Notably, it appears that there are additive genetic effects of the genotype of rs2908289 in the *GCK* gene on log-UACR. In a study on 42 families with maturity onset diabetes of the young 2 conducted in France, it was found that proteinuria develops at a relatively low frequency of 6% among these individuals, and another study showed similar results [27, 31]. On the other hand, functional and structural types of renal damage develop in GCK knockout mice according to several experimental studies [32, 33]. In a study on the Japanese population, GCK regulatory protein polymorphism was found to be significantly associated with the risk of chronic kidney disease (CKD) [34]. Recently, Böger et al. found that a common variant of the gene encoding a GCK-regulatory protein, a major cellular determinant of GCK enzymatic activity, may have a protective effect against T2D and CKD [35, 36]. Furthermore, Li et al. demonstrated that downregulation of GCK can contribute to the development of diabetic cardiomyopathy via increased oxidative stress and insulin resistance in an experimental animal model. Lastly, Szopa et al. reported that flow-mediated vasodilatation of the brachial artery is decreased in *GCK* mutation carriers [37, 38]. Such findings suggest that genetic variation in the *GCK* gene may exert positive or negative effect(s) not only on glucose metabolism in the liver and pancreas but also on the cardiovascular protective mechanism. Along with these studies, our findings of additive effects of the genotype of rs2908289 in the *GCK* gene on microalbuminuria should be considered in the future research on genetic interactions in complex trait variation.

In this study, we found that a novel SNP of isolated prediabetes, rs917793 in the *YKT6* gene, is related to the development of microalbuminuria. YKT6 is a soluble N-ethylmaleimide-sensitive factor attachment protein receptor (SNARE) that participates in the production and release of exosomes, such as synaptic-vesicle exocytosis. *YKT6* expression may be associated with the survival of cancer cells in lung and breast tissue [39, 40], but how genetic polymorphisms of the *YKT6* gene may exert positive or negative effects on glucose homeostasis and on related complications is still poorly understood.

There are some limitations of this study. First, to efficiently analyze the potential nongenetic factors associated with hyperglycemia or microalbuminuria and to obtain consistent results, we excluded many participants with DM or other chronic medical diseases related to microalbuminuria. Such a study design resulted in a relatively small sample size for the GWAS. Nevertheless, considering that this study was community-based and that no participants had any medical illness, the sample size in this GWAS is large enough to show a relation between microalbuminuria and genetic factors of prediabetes. Second, a social desirability bias cannot be ruled out because the medical history and the use of medication, tobacco, or alcohol were all self-reported by the subjects. This approach may have contributed to the discrepancies with other studies. Third, although we performed urinary dipstick tests, we could not completely rule out an asymptomatic urinary tract infection because urine culture analysis was not performed. Fourth, the test for albuminuria was conducted only during the first year of the study; therefore, it was impossible to obtain information about how many microalbuminuric patients progress to macroalbuminuria and DKD. Finally, because of population differences in allelic heterogeneity, generalization of the findings to all populations remains uncertain. Nonetheless, genetic studies on prediabetes in Asian populations may not necessarily confirm the same arrangement of susceptibility genes as those in other ethnic populations.

Despite the limitations, our results suggest that prediabetes may have a genetic impact on the development of diabetic complications. Therefore, genetic testing and early diagnosis of prediabetes can enable accurate prediction of diabetic complications, long-term health monitoring such as UACR for the subjects at a genetic risk and their families, and a preventive strategy against inevitable consequences such as DKD and end-stage renal disease. Furthermore, the genetic information associated with prediabetes can be useful for designing personalized treatments and for the development of new drugs for more precise medical care.

In conclusion, the results of the present study show a significant association between prediabetes and genetic polymorphisms of the *GCK* gene. Our results also revealed that SNP rs2908209 in the *GCK* gene can predict a lifelong risk of renal microvascular complications associated with prediabetes. We believe that genetic epidemiological studies of such associations may help to uncover the genetic basis of hyperglycemia-associated complications. Further research on the genetic factors influencing the development of albuminuria would be worthwhile because early detection and management of at-risk patients should help to inhibit the development and progression of CKD.

## Materials and methods

### Study population

This cross-sectional study on two population-based cohorts from Ansan and Ansung, Korea, was conducted by the Korean National Institute of Health as part of the KoGES, a Korean government-funded epidemiological survey to investigate trends in chronic diseases [41, 42]. All the participants volunteered and provided written informed consent prior to their enrollment. All the participants’ records, excluding the survey date and home region, were anonymized and deidentified before analysis by the authors. This study’s protocol was approved by the Institutional Review Board (IRB) of the Korea Centers for Disease Control and Prevention (IRB: 2014-10CON-06-P-E).

A total of 10,038 individual participants were examined biannually using laboratory tests, electrocardiograms, chest X-rays, and health questionnaires, and a 10-year follow-up study was recently completed. An oral glucose tolerance test was performed using the blood samples collected after fasting and 120 min after ingestion of 75 g of glucose. To clearly identify possible risk factor(s) for microvascular complications, the participants with a history of DM, essential hypertension, cancer, previously known kidney disease, or a UACR > 300 mg/(g creatinine) were excluded from this study (S1 Fig). Participants with urinary tract infection were excluded from the study on the basis of urinalysis. According to the 2016 American Diabetes Association standards of medical care, prediabetes is defined as aberrant fasting glucose (fasting plasma glucose between 5.55 and 6.94 mmol/L), impaired glucose tolerance (2-hr plasma glucose between 7.77 and 11.04 mmol/L), or HbA1c in the range 5.7–6.4% [5]. All eligible participants were subdivided into two groups: healthy controls and participants with prediabetes. Microalbuminuria was measured using spot morning urine and defined as a UACR between 30 and 300 mg/(g creatinine).

### Genotyping

We analyzed the data on SNPs using publicly available whole-genome data from the Korea association resource (KARE) project from KoGES, and used the Affymetrix Genome-Wide Human SNP Array 5.0 (Affymetrix Inc., Santa Clara, CA, USA) to genotype the samples from the Ansan and Ansung cohorts. The Bayesian robust linear model with the Mahalanobis distance algorithm was used to determine the genotypes of each SNP. SNPs were excluded if any of the following criteria were met: (1) a call rate lower than 95%, (2) a minor allele frequency below 0.05, or (3) a significant deviation from Hardy–Weinberg equilibrium below 0.001. Among the SNPs filtered by these criteria, only tagging SNPs were used for analysis here.

### Statistical analysis

Results are expressed as mean ± SD or as frequencies (and proportions). The normality of the distribution of parameters was analyzed by the Kolmogorov-Smirnov test. If a variable did not follow a normal distribution, a natural logarithm transformation was applied before statistical analysis. Because distribution of UACR was skewed to the right, this variable was log-transformed for further statistical analysis. Student’s *t* test was used to evaluate the differences in means between the two groups, or one-way analysis of variance was used for more than two groups. Categorical variables were assessed using chi-square analysis with Fisher’s exact test when the number of data points was small.

We performed linkage disequilibrium analysis of the analyzed polymorphisms of susceptibility genes using the Haploview software version 4.1 and generated a regional association analysis using HapMap, a web-based tool for identification and annotation of proxy SNPs. Mendelian randomization was applied to examine the direction of causality between microalbuminuria and the candidate SNP of susceptibility to prediabetes. We compared the results from the instrumental variable estimates of the association between genetic variation(s) and phenotypic measures to those from the least-squares linear regression using the Durbin form of the Durbin–Wu–Hausman statistic [43, 44]. Odds ratios (ORs) with 95% CIs were calculated using multiple logistic regression models according to UACR (control vs. microalbuminuria). To identify age-adjusted effects of significant and suggestive SNPs, we performed analysis of covariance using the least significant difference method. All statistical analyses were conducted in the PLINK software, version 1.09, or Statistical Analysis Software (version 9.3; SAS Institute Inc., Cary, NC, USA).

## Supporting information

S1 Fig A flow chart of the study group enrollment process. (TIF)

S2 Fig A quantile plot of the observed p values (black dots) for the association of all 1,198,063 SNPs at the GWAS stage. (TIF)

S1 Table Genotype distribution of the top 20 single nucleotide polymorphisms (SNPs) associated with a prediabetic state in the Ansung cohort. (DOC)

S2 Table Genotype distribution of SNPs associated with a prediabetic state in the Ansan cohort. (DOC)
