# Supplementary figures and images for: Association of prediabetes-associated single nucleotide polymorphisms with microalbuminuria
Source: PLoS One. 2017 Feb 3;12(2):e0171367. doi: 10.1371/journal.pone.0171367 (PMC5291388; doi:10.1371/journal.pone.0171367)

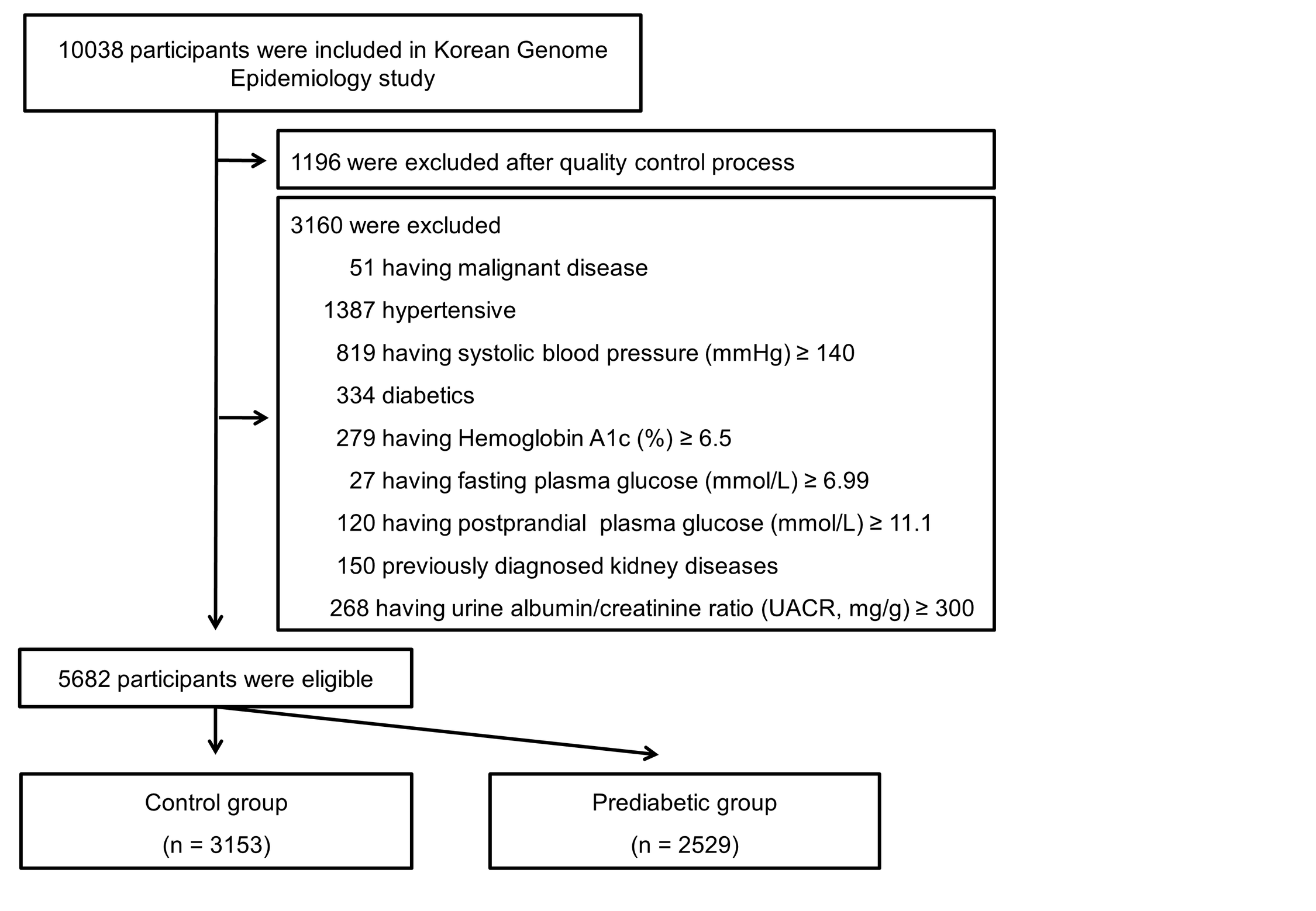

Supplement: S1 Fig — (TIF) [file pone.0171367.s001.tif]

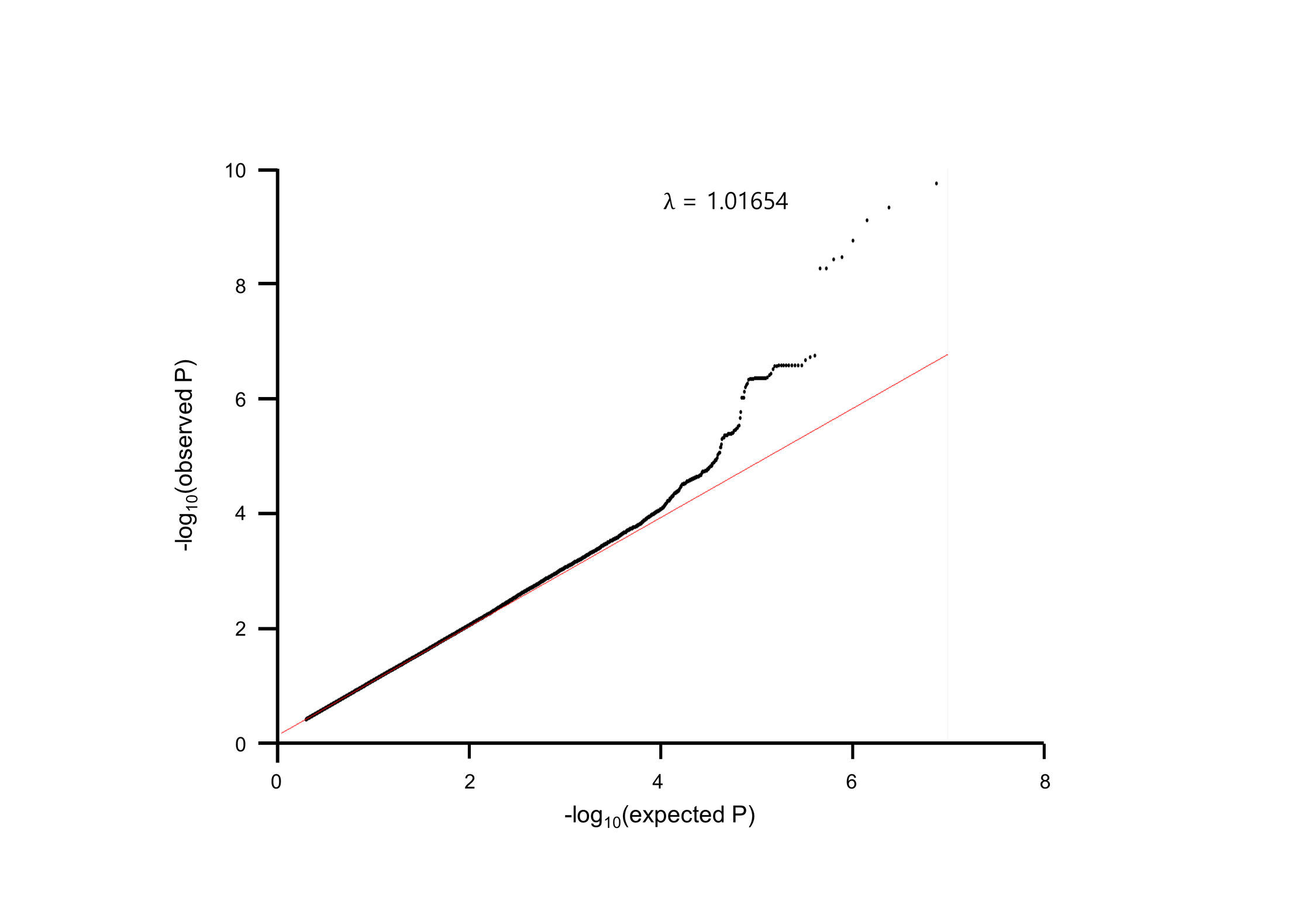

Supplement: S2 Fig — (TIF) [file pone.0171367.s002.tif]
