# Supplementary material for: Association of prediabetes-associated single nucleotide polymorphisms with microalbuminuria
Source: PLoS One. 2017 Feb 3;12(2):e0171367. doi: 10.1371/journal.pone.0171367 (PMC5291388; doi:10.1371/journal.pone.0171367)
Supplement: S1 Table — (DOC) [file pone.0171367.s003.doc]

**Table S1**. Genotype distribution of the top 20 single nucleotide polymorphisms (SNPs) associated with a prediabetic state in the Ansung cohort

| dbSNP ID | CHR | Position | Risk |  | Additive* | |  | Dominant* | |  | Recessive* | |  | Nearest gene |
| --- | --- | --- | --- | --- | --- | --- | --- | --- | --- | --- | --- | --- | --- | --- |
|  |  |  | allele |  | OR | p |  | OR | p |  | OR | p |  |  |
| rs1572037 | 1 | 3337805 | T |  | 1.220 | 9.33 x 10^-4^ |  | 1.477 | 3.06 x 10^-5^ |  | 1.155 | 1.52 x 10^-1^ |  | PRDM16 |
| rs7539624 | 1 | 223719125 | G |  | 0.805 | 3.02 x 10^-4^ |  | 0.691 | 9.02 x 10^-5^ |  | 0.806 | 3.35 x 10^-2^ |  | CAPN2 |
| rs6722447 | 2 | 127494658 | G |  | 0.650 | 1.02 x 10^-4^ |  | 0.897 | 2.02 x 10^-1^ |  | 0.425 | 9.58 x 10^-5^ |  | IWS1 |
| rs12621149 | 2 | 127509268 | A |  | 0.649 | 9.50 x 10^-5^ |  | 0.889 | 1.66 x 10^-1^ |  | 0.425 | 9.63 x 10^-5^ |  | IWS1 |
| rs495074 | 2 | 168830831 | C |  | 1.237 | 4.20 x 10^-4^ |  | 1.138 | 1.55 x 10^-1^ |  | 1.524 | 6.24 x 10^-5^ |  | NOSTRIN |
| rs2052975 | 2 | 225486005 | A |  | 1.275 | 8.19 x 10^-5^ |  | 1.236 | 1.50 x 10^-2^ |  | 1.521 | 1.66 x 10^-4^ |  | NYAP2 |
| rs3791419 | 2 | 239113682 | C |  | 0.862 | 2.04 x 10^-2^ |  | 1.150 | 1.07 x 10^-1^ |  | 0.632 | 8.74 x 10^-5^ |  | HDAC4 |
| rs3184121 | 3 | 124967889 | C |  | 0.813 | 2.63 x 10^-3^ |  | 0.715 | 5.32 x 10^-5^ |  | 0.773 | 4.92 x 10^-2^ |  | HEG1 |
| rs9682173 | 3 | 127535196 | T |  | 0.821 | 7.18 x 10^-3^ |  | 0.724 | 9.38 x 10^-5^ |  | 0.777 | 7.48 x 10^-2^ |  | LINC01471 |
| rs1542567 | 3 | 140355940 | G |  | 1.648 | 1.98 x 10^-4^ |  | 0.935 | 4.62 x 10^-1^ |  | 2.852 | 8.77 x 10^-5^ |  | CLSTN2 |
| rs6787578 | 3 | 159498481 | C |  | 0.982 | 9.45 x 10^-1^ |  | 0.647 | 8.65 x 10^-5^ |  | 1.042 | 9.38 x 10^-1^ |  | IQCJ-SCHIP1/  SCHIP1 |
| rs1387696 | 3 | 159527474 | G |  | 0.778 | 4.98 x 10^-1^ |  | 0.568 | 6.99 x 10^-6^ |  | 0.650 | 5.60 x 10^-1^ |  | IQCJ-SCHIP1/  SCHIP1 |
| rs10036189 | 5 | 115995373 | C |  | 2.934 | 5.06 x 10^-2^ |  | 1.622 | 9.54 x 10^-5^ |  | 8.092 | 5.76 x 10^-2^ |  | LVRN |
| rs6900694 | 6 | 16743461 | T |  | 0.788 | 1.98 x 10^-4^ |  | 0.684 | 1.02 x 10^-5^ |  | 0.747 | 1.41 x 10^-2^ |  | ATXN1 |
| rs9356748 | 6 | 20724866 | T |  | 0.831 | 1.78 x 10^-3^ |  | 0.691 | 6.51 x 10^-5^ |  | 0.878 | 1.92 x 10^-1^ |  | CDKAL1 |
| rs7747752 | 6 | 20725192 | G |  | 0.832 | 1.83 x 10^-3^ |  | 0.689 | 5.54 x 10^-5^ |  | 0.882 | 2.10 x 10^-1^ |  | CDKAL1 |
| rs17182834 | 6 | 113634170 | T |  | 0.841 | 9.71 x 10^-2^ |  | 0.705 | 4.74 x 10^-5^ |  | 0.800 | 2.81 x 10^-1^ |  | LOC101927686 |
| rs2908289 | 7 | 44184343 | A |  | 1.245 | 4.40 x 10^-2^ |  | 1.440 | 2.93 x 10^-5^ |  | 1.388 | 1.29 x 10^-1^ |  | GCK/  LOC105375257 |
| rs1799884 | 7 | 44189469 | T |  | 1.245 | 4.46 x 10^-2^ |  | 1.430 | 3.96 x 10^-5^ |  | 1.389 | 1.29 X 10^-1^ |  | GCK |
| rs917793 | 7 | 44206254 | A |  | 1.307 | 5.19 x 10^-3^ |  | 1.483 | 4.29 x 10^-6^ |  | 1.502 | 3.11 X 10^-2^ |  | YKT6 |

*calculated by logistic regression analysis with age and gender as covariates
