# Supplementary material for: Association of prediabetes-associated single nucleotide polymorphisms with microalbuminuria
Source: PLoS One. 2017 Feb 3;12(2):e0171367. doi: 10.1371/journal.pone.0171367 (PMC5291388; doi:10.1371/journal.pone.0171367)
Supplement: S2 Table — (DOC) [file pone.0171367.s004.doc]

**Table S2**. Genotype distribution of SNPs associated with a prediabetic state in the Ansan cohort

| dbSNP ID | CHR | Position | Risk |  | Additive* | |  | Dominant* | |  | Recessive* | |  | Nearest gene |
| --- | --- | --- | --- | --- | --- | --- | --- | --- | --- | --- | --- | --- | --- | --- |
|  |  |  | allele |  | OR | p |  | OR | p |  | OR | p |  |  |
| rs1572037 | 1 | 3337805 | T |  | 0.999 | 9.91 x 10^-1^ |  | 1.008 | 9.23 x 10^-1^ |  | 0.992 | 9.24 x 10^-1^ |  | PRDM16 |
| rs7539624 | 1 | 223719125 | G |  | 1.027 | 5.99 x 10^-1^ |  | 1.057 | 4.97 x 10^-1^ |  | 1.019 | 8.31 x 10^-1^ |  | CAPN2 |
| rs6722447 | 2 | 127494658 | G |  | 1.080 | 3.55 x 10^-1^ |  | 0.889 | 1.10 x 10^-1^ |  | 1.238 | 1.93 x 10^-1^ |  | IWS1 |
| rs12621149 | 2 | 127509268 | A |  | 1.085 | 3.29 x 10^-1^ |  | 0.890 | 1.11 x 10^-1^ |  | 1.248 | 1.78 x 10^-1^ |  | IWS1 |
| rs495074 | 2 | 168830831 | C |  | 1.123 | 2.74 x 10^-2^ |  | 1.175 | 4.58 x 10^-2^ |  | 1.162 | 9.77 x 10^-2^ |  | NOSTRIN |
| rs2052975 | 2 | 225486005 | A |  | 1.085 | 1.43 x 10^-1^ |  | 1.024 | 7.57 x 10^-1^ |  | 1.190 | 8.69 x 10^-2^ |  | NYAP2 |
| rs3791419 | 2 | 239113682 | C |  | 1.043 | 4.40 x 10^-1^ |  | 1.070 | 3.72 x 10^-1^ |  | 1.049 | 6.26 x 10^-1^ |  | HDAC4 |
| rs3184121 | 3 | 124967889 | C |  | 1.130 | 4.39 x 10^-2^ |  | 1.080 | 2.91 x 10^-1^ |  | 1.253 | 4.90 x 10^-2^ |  | HEG1 |
| rs9682173 | 3 | 127535196 | T |  | 0.994 | 9.20 x 10^-1^ |  | 1.024 | 7.44 x 10^-1^ |  | 0.973 | 8.17 x 10^-1^ |  | LINC01471 |
| rs1542567 | 3 | 140355940 | G |  | 1.009 | 9.32 x 10^-1^ |  | 1.019 | 8.13 x 10^-1^ |  | 1.014 | 9.51 x 10^-1^ |  | CLSTN2 |
| rs6787578 | 3 | 159498481 | C |  | 1.102 | 6.27 x 10^-1^ |  | 0.853 | 1.03 x 10^-1^ |  | 1.249 | 5.77 x 10^-1^ |  | IQCJ-SCHIP1/  SCHIP1 |
| rs1387696 | 3 | 159527474 | G |  | 0.838 | 5.34 x 10^-1^ |  | 0.945 | 5.99 x 10^-1^ |  | 0.706 | 5.41 x 10^-1^ |  | IQCJ-SCHIP1/  SCHIP1 |
| rs10036189 | 5 | 115995373 | C |  | 0.735 | 3.76 x 10^-1^ |  | 0.969 | 7.73 x 10^-1^ |  | 0.541 | 3.78 x 10^-1^ |  | LVRN |
| rs6900694 | 6 | 16743461 | T |  | 0.946 | 3.31 x 10^-1^ |  | 0.948 | 4.70 x 10^-1^ |  | 0.912 | 3.89 x 10^-1^ |  | ATXN1 |
| rs9356748 | 6 | 20724866 | T |  | 0.847 | 1.23 x 10^-3^ |  | 0.845 | 3.89 x 10^-2^ |  | 0.762 | 1.58 x 10^-3^ |  | CDKAL1 |
| rs7747752 | 6 | 20725192 | G |  | 0.847 | 1.23 x 10^-3^ |  | 0.846 | 3.98 x 10^-2^ |  | 0.762 | 1.53 x 10^-3^ |  | CDKAL1 |
| rs17182834 | 6 | 113634170 | T |  | 0.953 | 6.01 x 10^-1^ |  | 1.142 | 7.46 x 10^-2^ |  | 0.858 | 3.98 x 10^-1^ |  | LOC101927686 |
| rs2908289 | 7 | 44184343 | A |  | 1.572 | 8.97 x 10^-6^ |  | 1.283 | 1.05 x 10^-3^ |  | 2.337 | 2.66 x 10^-5^ |  | GCK/  LOC105375257 |
| rs1799884 | 7 | 44189469 | T |  | 1.575 | 8.11 x 10^-6^ |  | 1.298 | 6.07 x 10^-4^ |  | 2.337 | 2.65 x 10^-5^ |  | GCK |
| rs917793 | 7 | 44206254 | A |  | 1.396 | 9.79 x 10^-5^ |  | 1.281 | 9.34 x 10^-4^ |  | 1.829 | 3.61 x 10^-4^ |  | YKT6 |

*calculated by logistic regression analysis with age and gender as covariate
